# Men in eating disorder units: a service evaluation survey regarding mixed gender accommodation rules in an eating disorder setting

**DOI:** 10.1192/bjb.2018.51

**Published:** 2018-12

**Authors:** Akira Fukutomi, Frances Connan, Anthony P. Winston, Pia Ghosh

**Affiliations:** 1 Vincent Square Eating Disorder Service, Central and North West London National Health Service Foundation Trust, UK; 2 The Aspen Centre, Coventry and Warwickshire Partnership National Health Service Trust, UK

**Keywords:** Eating disorders, mixed gender accommodation, Department of Health, Care Quality Commission, CQC, Anorexia Nervosa

## Abstract

**Aims and method:**

This service evaluation was conducted to find out: (1) if mixed gender accommodation in eating disorder units is perceived to be helpful or unhelpful for recovery, and (2) if men were being discriminated against by the implementation of the 2010 Department of Health (DoH) guidelines on the elimination of mixed gender wards. All 32 in-patient units accredited on the Quality Network for Eating Disorders were contacted via a survey.

**Results:**

We received 38 responses from professionals from 26 units and 53 responses from patients (46 female, 7 male) from 7 units. Four units had closed admissions to male patients due to DoH guidelines.

**Clinical implications:**

We found that it is possible to provide admission for men with eating disorders, while respecting the single gender accommodation rules, and that doing so is likely to be helpful for both genders and prevents discrimination against men.

**Declaration of interest:**

None.

In 2010, the Department of Health (DoH) set guidelines for all hospitals to eliminate mixed gender wards to preserve privacy and dignity for patients.^1^ In specialist eating disorder services, all-male wards do not exist due to the low prevalence of the disorder in males compared with females. This leads to the paradoxical situation where men have difficulty accessing an eating disorder bed under these DoH guidelines. Some hospital trusts have modified their accommodation or procedures in light of Care Quality Commission (CQC) guidelines set by the DoH to accommodate men; however some have had to close admissions for male patients as the trusts believed they would be fined for breaching single gender accommodation rules.

Anecdotally, there has been positive feedback from having men in eating disorder wards: they provide a less competitive atmosphere, a different perspective in group treatments and reduce the institutional nature of the environment. A survey performed by Mezey *et al*^2^ in a medium secure forensic setting found similar results, where most female patients preferred to be among male patients. A small qualitative study interviewed male in-patients with eating disorders and one participant said that eating disorder is ‘gender-excluding as a disorder’ and ‘reasons why you get there are probably slightly different but in the end all roads lead to Rome’,^3^ suggesting that the same treatment applies to both genders. There has yet to be any evaluation of patient perceptions on mixed versus single gender environments in an eating disorder setting. We therefore collaborated with National Health Service England to conduct a service evaluation survey, gathering information from patients and professionals.

## Method

All 32 in-patient units accredited on the Quality Network for Eating Disorders (QED) in the UK were contacted via email, using an electronic survey (Supplementary Appendix 1 available at https://doi.org/10.1192/bjb.2018.51). The individuals contacted were those registered on the QED network and included consultant psychiatrists, psychologists, senior nurses and occupational therapists. The survey for the professionals asked for feedback on three themes:

(1) if they were a single or mixed gender unit and how they arranged the ward to accommodate both genders;
(2) if the trust, commissioners or the CQC had ever commented on or stopped admission of male patients;
(3) if the professional had any views on recovery of patients in same or mixed gender environments.

The third point was conducted by asking positively and negatively framed questions with a five-point Likert scale and mean scores were then calculated. The points ranged from 1 for strongly disagree to 5 for strongly agree. A ‘free comment’ box was placed at the end for added opinions.

In-patients were asked to provide feedback on their experience of single or mixed gender environments and similar scores were then calculated (Supplementary Appendix 2). Male patients had extra questions to complete. Data were collected between October 2016 and August 2017.

## Results

### Professionals

**Fig. 1:**
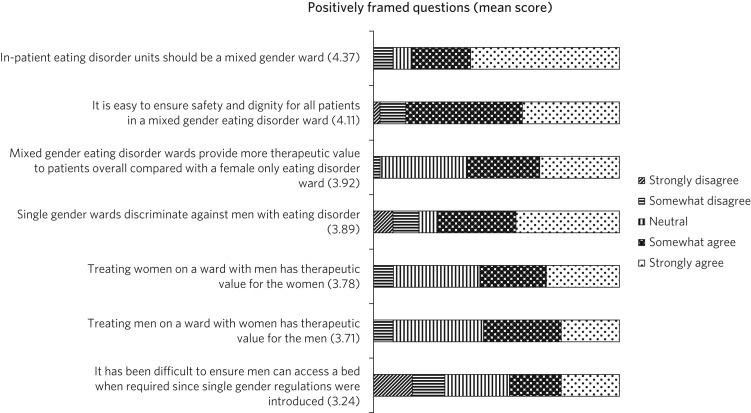
Survey for professionals: positively framed questions.

**Fig. 2:**
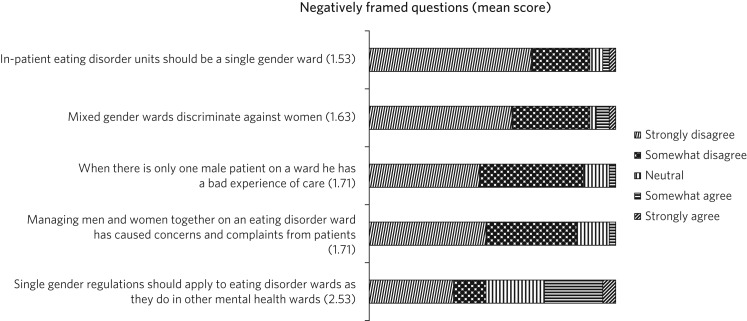
Survey for professionals: negatively framed questions.

A total of 38 eating disorder professionals registered on the QED network responded from 26 different units across the UK. Six of these units did not admit men: three of these units had stopped admitting men after a CQC inspection and one had been advised similarly by Health Improvement Scotland. Figures 1 and 2 show the responses to questions on perceptions of mixed gender wards. In general, professionals thought that mixed gender units carried more benefit than harm to patients. There was a general agreement that eating disorder units should be mixed gender wards (mean score 4.37 out of 5) and that it was easy to ensure safety and dignity in mixed gender wards (4.11). Most did not think that mixed gender wards discriminated against women (1.63), nor did they think that having one male patient was detrimental to that man's care (1.71). There were mixed responses about whether it has been difficult to find a bed for male patients since the single gender rule was introduced (3.24). Table 1 highlights some comments that were provided in the free-text box.

**Table 1:** Comments by professionals in the free-text box

Themes	Response from professionals
Is mixed gender accommodation helpful in eating disorder units?	‘On the whole we have found having a male on the unit quite refreshing and beneficial to both client sexes…We have only had a small number of males admitted to the unit but on the whole our experience so far is positive.’ ‘Mixed sex units for ED are a necessity. Both sexes benefit from having the other gender on the ward. ED units attempt to support individuals in recognising what is “normal” this includes being in an environment with members of the opposite sex.’ ‘We tailor our groups to accommodate the needs of all patients, and dynamics that arise around gender, as with any other issue, would be explored through the group process and in community group settings where appropriate.’
Are men being discriminated against due to single gender accommodation guidelines?	‘The concern seems disproportionate and the impression is that the response from CQC varies dependent upon the individual assessor on the day.’ ‘I believe it could be quite tricky to manage the issue of having different corridors for male and females especially if you are not a purpose built unit. The feedback from males during their stay is they prefer to be included in the group as it often makes them feel awkward and isolated from peers.’ ‘There needs to be some work done to alleviate blanket rules around single sex wards. Some elements ought to be ward or unit specific.’

### Patients

A total of 53 patients (46 female, 7 male) from 7 eating disorder units responded to the survey. Three of these surveys were partially incomplete; however the scores and comments that were provided have been included in the results. A total of 49 participants (92%) had experienced admission on a mixed gender unit and 29 (56%) had experienced both single and mixed gender environments. Table 2 shows the distribution of services from which the patients responded, although many had experience of admission at a variety of other centres.

**Table 2:** Sources of responses

Eating disorder unit	Number of responses
Vincent Square Eating Disorder Service, London	23
The Haldon Eating Disorder Service, Exeter	15
The Priory Hospital, Chelmsford	9
Specialist Treatment for Eating Problems (STEPs), Bristol	3
Kimmeridge Court, Dorset Healthcare Services, Dorset	1
The Retreat, York	1
West Park Hospital, Northern Centre for Eating Disorders, Durham	1
Total	53

**Fig. 3:**
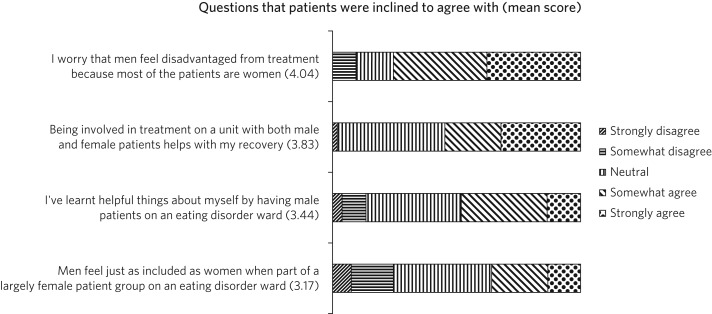
Patients’ responses scoring above 3 (neutral).

**Fig. 4:**
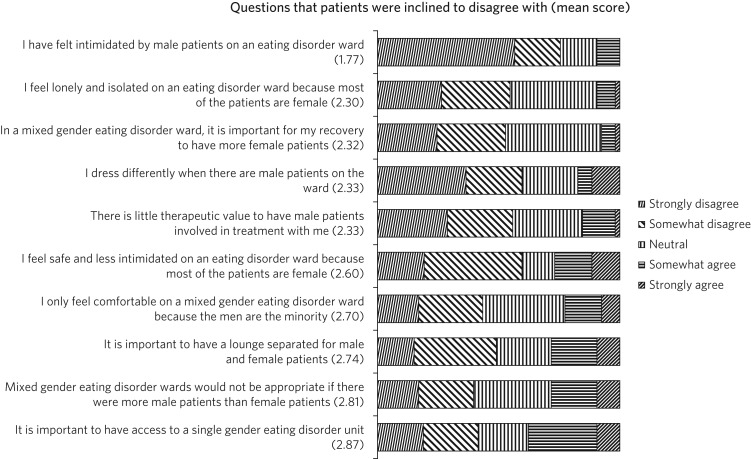
Patients’ responses scoring below 3 (neutral).

Figures 3 and 4 shows the attitudes from patients towards mixed gender accommodation. Most patients agreed that men were being disadvantaged (75.5%). Nearly the entire patient group (98%) answered positively or neutrally to whether having a mixed gender accommodation was helpful for their recovery. A total of 45 participants (85%) gave a similar (positive or neutral) response to ‘I've learnt helpful things about myself by having male patients’. There were minimal safety issues noted, most participants (75.5%) disagreed to being ‘intimidated by male patients on the ward’.

**Fig. 5:**
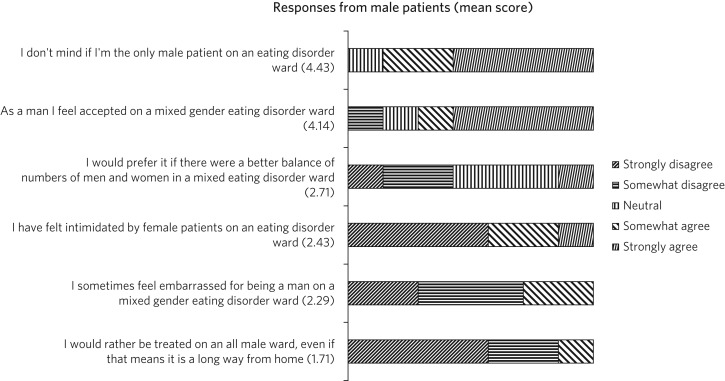
Perceptions from male patients.

Figure 5 shows the responses from the seven male patients. Of these, six agreed to the statement ‘I don't mind if I'm the only male patient on an eating disorder ward’ and five agreed to ‘As a man I feel accepted on a mixed gender ward’. Six men said that they would not want to be treated on an all-male ward if it were far from home.

Of the 41 patients who wrote in the optional free-text box (see Table 3), 36 (88.8%) were in favour of having a mixed gender unit. Many voiced that having a ‘mixed unit is reflective of the outside world’ and that mixed wards were ‘healthy for dynamics’ and ‘reduces competitiveness’. A male patient thought it was ‘crucial’ to his recovery that he was on a mixed gender ward and believes his presence ‘was a benefit to others on the ward’. Only three participants (8%) had negative feelings towards this (one male, two female); however both female patients with this opinion had not experienced a mixed gender eating disorder ward. The male patient voiced strong concerns about feeling ‘isolated and slightly intimidated’ as the only man because ‘many groups were geared towards females’.

**Table 3:** Comments by patients in the free-text box

Themes	Response from patients
In favour of having mixed gender wards	‘Mixed unit is reflective of the outside world.’ ‘Having a male upon the ward allowed me to see from a different perspective during therapy groups which helped me to understand the illness from this kind of viewpoint and hence supported my treatment.’ ‘All female wards can be stifling and very competitive.’ ‘I feel sure that had I been forced to be on an all-male ward that I would have found it much harder. I also believe that my presence as a man was a benefit to the others on the ward.’ ‘I think it's not a problem having male patients on the ward too. It's not fair to restrict their treatment options.’
In favour of having single gender wards	‘I see that there will be fewer wards for men which is unfair but also there is a percentage of women with eating disorders who have PTSD or have suffered from sexual abuse that need to be taken into account.’ ‘Many groups were very geared toward females and I have often felt isolated and slightly intimidated in both units. I have often felt that my recovery, or lack thereof, has been in part not helped by being the only male in treatment at in-patient units, although I would still rather be the only male than have no treatment or community based treatment only which for me is ineffective.’

## Discussion

The results show that the majority of both patients and staff believe mixed gender units work just as well as, if not better than, single gender units for patient recovery in both genders. Some of the original thoughts behind segregating the genders, such as having to dress differently or having separate lounges, were not deemed very important by the patients. Unfortunately we confirmed that a handful of units have closed to male admissions due to interpretation of DoH regulations. Drawing parallels from the study by Mezey *et al*,^2^ it may be that due to the long-stay nature of the ward and treatment duration, having a sense of normality and the reduction in competitiveness by having a mixed gender unit helps to provide a more therapeutic setting. This survey suggests that both professionals and patients believe that eating disorder wards should be open to males, and that patients will have a better recovery journey as a result of the mixed gender environment. We hope that research will now follow to better understand the risks and values of a mixed gender treatment environment.

## Recommendations

With the support of QED, clarity was sought about how the DoH single gender guidelines apply to settings such as eating disorder services. The following guidance has been approved by the CQC as entirely consistent with the DoH single gender accommodation guidance and should make possible male admissions to every in-patient eating disorder service:

• A risk assessment has been carried out to ensure that the male patient does not pose a specific risk to female patients.
• There is an agreement in place with National Health Service England commissioners on the admission of male patients and the admission is consistent with this agreement.
• Appropriate arrangements have been put in place to ensure that female patients do not feel unsafe or compromised in terms of privacy.
• Male patients are accommodated in single bedrooms with en-suite bathroom and toilet facilities, if possible.
• If this is not possible, male patients occupy a single room with use of male-only bathroom and toilet facilities.
• Patients do not have to walk through a sleeping area or a bathroom occupied by another gender. A sleeping area is a bedroom or a bay of beds. Men can walk through a corridor, off of which there are doors to female bedrooms and bathrooms, to access a male bathroom.
• A women-only day room is available.

## About the authors

**Akira Fukutomi** is in Core Training Year 3 at Vincent Square Eating Disorder Service, Central and North West London National Health Service Foundation Trust, UK. **Frances Connan** is a Consultant Psychiatrist at Vincent Square Eating Disorder Service, Central and North West London National Health Service Foundation Trust, UK. **Anthony P. Winston** is a Consultant Psychiatrist in Eating Disorders at The Aspen Centre, Coventry and Warwickshire Partnership National Health Service Trust, Warwick, UK. **Pia Ghosh** is in Speciality Training Year 4 at Vincent Square Eating Disorder Service, Central and North West London National Health Service Foundation Trust, UK.

## Supplementary material

For supplementary material accompanying this paper visit http://dx.doi.org/10.1192/bjb.2018.51. click here to view supplementary material
