# Supplementary material for: Men in eating disorder units: a service evaluation survey regarding mixed gender accommodation rules in an eating disorder setting
Source: BJPsych Bull. 2018 Dec;42(6):258–63. doi: 10.1192/bjb.2018.51 (PMC6465224; doi:10.1192/bjb.2018.51)
Supplement: Supplementary file 1 [file S2056469418000517sup001.docx]

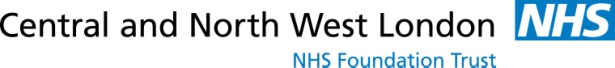
Appendix 1: QED Eating Disorder Service Questionnaire Re: Mixed Sex Wards for professionals

**1. Name of Service**

**2. Do you currently admit male patients?**

Yes

No - please skip to question 7

**3. How many beds does your unit have and how many can be used for men?**

Total bed number

Maximum male bed number

**4. Does your ward have the following facilities arranged to cater for a mixed ward environment?**

|  | Yes | No |
| --- | --- | --- |
| **Separate toilets for male and female patients** |  |  |
| **Separate shower for male and female patients** |  |  |
| **Separate corridor for male and female patient's bedrooms** |  |  |
| **Male only lounge** |  |  |
| **Female only lounge** |  |  |
| **Mixed sex lounge** |  |  |
| **All bedrooms ensuite** |  |  |
| **All male rooms ensuite** |  |  |
| **Some male rooms ensuite** |  |  |
| **All female rooms ensuite** |  |  |
| **Some female rooms ensuite** |  |  |

**5. Are the single sex rules on the facilities strictly followed by patients?**

|  | *Yes* | *No* | *N/A* |
| --- | --- | --- | --- |
| *Toilets* |  |  |  |
| *Shower* |  |  |  |
| *Lounge* |  |  |  |

**6. Please describe your ward arrangement to cater for a mixed sex ward and how you ensure that the ward environment meets all the criteria for single sex accommodation with CQC guidelines. Please include the details of how the male/female bedrooms/washrooms are separated, including corridors/floors/doors**

**7. Has your unit been stopped from admitting patients in the past?**

No

Yes - please specify reason (e.g by CQC or Trust)

**8. Has another organisation (e.g CQC) ever commented or taken action on the sex separation arrangement of the ward? If yes, please expand on who and what they commented on.**

No

Yes - please specify

**9. Please tick on how much you agree or disagree with these following statements:**

|  | *Strongly Disagree* | *Somewhat Disagree* | *Neutral* | *Somewhat Agree* | *Strongly Agree* |
| --- | --- | --- | --- | --- | --- |
| *Inpatient ED units should be a mixed sex ward* |  |  |  |  |  |
| *Mixed sex wards discriminate against women* |  |  |  |  |  |
| *It has been difficult to ensure men can access a bed when required since single sex regulations were introduced* |  |  |  |  |  |
| *It is easy to ensure safety and dignity for all patients in a mixed sex ED ward* |  |  |  |  |  |
| *When there is only one male patient on a ward he has a bad experience of care* |  |  |  |  |  |
| *Mixed sex ED wards provide more therapeutic value to patients overall compared to a female only ED ward* |  |  |  |  |  |
| *Treating women on a ward with men has therapeutic value for the women* |  |  |  |  |  |
| *Inpatient ED units should be a single sex ward* |  |  |  |  |  |
| *Treating men on a ward with women has therapeutic value for the male patients* |  |  |  |  |  |
| *Managing men and women together on an ED ward has caused concerns and complaints from patients* |  |  |  |  |  |
| *Single sex wards discriminate against men with ED* |  |  |  |  |  |
| *Single sex regulations should apply to ED wards as they do in other mental health wards* |  |  |  |  |  |

**10. Please leave any comments regarding the same-sex accommodation guideline in regards to eating disorder units.**

Bottom of Form

| 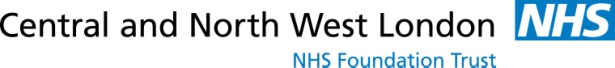Appendix 2: QED Eating Disorder Service Questionnaire Re: Mixed Sex Wards for Patients |
| --- |

**Top of Form**

**1. Name of eating disorder hospital / service**

**2. What is your gender?**

Female

Male

**3. Have you ever been admitted as an inpatient to an ED unit which had both male and female patients?**

Yes

No

**4. Have you had an admission or a period of an admission where only female patients were on the ED unit**

Yes

No

I am male and therefore not applicable

**5. All patients: Please rate how much you agree / disagree with these following statements:**

|  | **Strongly Disagree** | **Somewhat Disagree** | **Neutral** | **Somewhat Agree** | **Strongly Agree** |
| --- | --- | --- | --- | --- | --- |
| **Being involved in treatment on a unit with both male and female patients helps with my recovery** |  |  |  |  |  |
| **It is important to have access to a single sex ED unit** |  |  |  |  |  |
| **There is little therapeutic value to have male patients involved in treatment with me** |  |  |  |  |  |
| **It is important to have a lounge separated for male and women patients** |  |  |  |  |  |
| **In a mixed sex ED ward, it is important for my recovery to have more female patients** |  |  |  |  |  |
| **I've learnt helpful things about myself by having male patients on an ED ward** |  |  |  |  |  |
| **I feel lonely and isolated on an ED ward because most of the patients are female** |  |  |  |  |  |
| **I feel safe and less intimidated on an ED ward because most of the patients are female** |  |  |  |  |  |
| **Men feel just as included as women when part of a largely female patient group on an ED ward** |  |  |  |  |  |
| **Mixed sex ED wards would not be appropriate if there were more male patients than female patients** |  |  |  |  |  |
| **I dress differently when there are male patients on the ward** |  |  |  |  |  |
| **I only feel comfortable on a mixed sex ED ward because the men are the minority** |  |  |  |  |  |
| **I worry that men feel disadvantaged from treatment because most of the patients are female** |  |  |  |  |  |
| **I have felt intimidated by male patients on an ED ward** |  |  |  |  |  |

**6. Male patients only: Please rate how much you agree / disagree with the following statements as a male patient on an ED ward**

|  | **Strongly Disagree** | **Somewhat Disagree** | **Neutral** | **Somewhat Agree** | **Strongly Agree** |
| --- | --- | --- | --- | --- | --- |
| **As a man I feel accepted on a mixed sex ED ward** |  |  |  |  |  |
| **I would prefer it if there were a better balance of numbers of men and women in a mixed ED ward** |  |  |  |  |  |
| **I sometimes feel embarrassed for being a man on a mixed sex ED ward** |  |  |  |  |  |
| **I would rather be treated on an all male ward, even if that means it is a long way from home** |  |  |  |  |  |
| **I don't mind if I'm the only male patient on an ED ward** |  |  |  |  |  |
| **I have felt intimidated by female patients on a ED ward** |  |  |  |  |  |

**7. Once a decision to admit had been made with your team, how many days did you have to wait for a specialist eating disorder bed?**

days

**8. Please leave a comment about your thoughts on mixed sex ED units and how it affects your treatment**
